# SGLT2 Inhibitor Use and Cardiorenal Outcomes in Type 2 Diabetes With Liver Cirrhosis

**DOI:** 10.1001/jamanetworkopen.2025.60429

**Published:** 2026-02-23

**Authors:** Mu-Chi Chung, Tung-Min Yu, Laing-You Wu, Ming-Ju Wu, Jeng-Jer Shieh, Chi-Jung Chung

**Affiliations:** 1 Division of Nephrology, Department of Medicine, Taichung Veterans General Hospital, Taichung, Taiwan; 2 Division of Clinical Toxicology, Department of Medical Toxicology, Taichung Veterans General Hospital, Taichung, Taiwan; 3 Department of Post-Baccalaureate Medicine, College of Medicine, National Chung Hsing University, Taiwan; 4 Department of Public Health, College of Public Health, China Medical University, Taichung, Taiwan; 5 Institute of Biomedical Sciences, National Chung Hsing University, Taichung, Taiwan; 6 Department of Education and Research, Taichung Veterans General Hospital, Taichung, Taiwan; 7 Department of Medical Research, China Medical University Hospital, Taichung, Taiwan

## Abstract

**Importance:**

Type 2 diabetes (T2D) and liver cirrhosis frequently coexist, creating a high-risk population for adverse outcomes. Patients with both conditions face elevated risks of kidney and cardiovascular complications, yet evidence regarding optimal antidiabetic therapy in this vulnerable population remains limited.

**Objective:**

To evaluate the association of sodium-glucose cotransporter–2 inhibitor (SGLT2i) vs dipeptidyl peptidase–4 inhibitor (DPP4i) use with kidney outcomes, cardiovascular events, and hepatic decompensation in patients with concurrent T2D and liver cirrhosis.

**Design, Setting, and Participants:**

This nationwide retrospective cohort study utilized data from the Taiwan’s National Health Insurance Database between May 2016 and December 2023. Adults with both T2D and liver cirrhosis who initiated either SGLT2is or DPP4is were included.

**Exposure:**

Use of SGLT2is (dapagliflozin, empagliflozin, and canagliflozin) or DPP4is (alogliptin, linagliptin, sitagliptin, saxagliptin, and vildagliptin).

**Main Outcomes and Measures:**

The primary outcomes were end-stage kidney disease (ESKD), acute kidney injury (AKI), and major adverse cardiovascular events (MACE). Secondary outcomes included individual MACE components and hepatic decompensation events. Inverse probability of treatment weighting was employed to balance baseline characteristics.

**Results:**

Among 24 259 patients (mean [SD] age, 64.68 [11.95] years; 8229 female [33.92%]), 9689 (39.94) received SGLT2is and 14 570 (60.06%) received DPP4is. During a median (IQR) follow-up of 2.3 (1.0-4.0) years, compared with DPP4i use, SGLT2i treatment was associated with significantly reduced risks of ESKD (adjusted hazard ratio [HR], 0.34; 95% CI, 0.25-0.47), AKI (adjusted HR, 0.66; 95% CI, 0.59-0.74), and MACE (adjusted HR, 0.67; 95% CI, 0.62-0.71). Additionally, SGLT2i use was associated with a lower risk of all-cause mortality (adjusted HR, 0.58; 95% CI, 0.53-0.63) and hepatic decompensation events (adjusted HR, 0.65; 95% CI, 0.57-0.74).

**Conclusions and Relevance:**

In this cohort study of patients with T2D and liver cirrhosis, the use of SGLT2is was associated with substantially lower risks of adverse kidney, cardiovascular, and hepatic outcomes compared with DPP4is. These findings suggested significant cardiorenal and hepatic protection for SGLT2is in this high-risk population.

## Key Points

**Question:**

Is the use of sodium-glucose cotransporter–2 inhibitors (SGLT2is) associated with improved cardiorenal and hepatic outcomes in patients with type 2 diabetes (T2D) and liver cirrhosis?

**Findings:**

In this cohort study of 24 259 patients with T2D and cirrhosis, SGLT2is were associated with decreased risk of end-stage kidney disease, acute kidney injury, major adverse cardiovascular events, and all-cause mortality during a median follow-up of 2.3 years. SGLT2is were also associated with reduced risk of hepatic decompensation events, with consistent results across sensitivity and subgroup analyses.

**Meaning:**

These findings suggest that SGLT2is may provide significant cardiorenal and hepatic protection for patients with coexisting T2D and liver cirrhosis.

## Teaser

This cohort study analyzes associations of sodium-glucose cotransporter–2 (SGLT2) vs dipeptidyl peptidase–4 inhibitor use with kidney outcomes, cardiovascular events, and hepatic decompensation in patients with concurrent type 2 diabetes (T2D) and liver cirrhosis.

## Introduction

Type 2 diabetes (T2D) and liver cirrhosis frequently coexist and share a bidirectional association. Approximately 31% of patients with cirrhosis have T2D,^1^ and those with diabetes are approximately twice as likely to develop cirrhosis.^2^ When T2D and liver cirrhosis are present, patients face substantially worse outcomes, including increased risks of kidney complications,^3^ hepatic decompensation, and mortality.^2^

Despite the well-established cardiorenal benefits of sodium-glucose cotransporter–2 inhibitors (SGLT2is) in patients with T2D, their role in individuals with liver cirrhosis remains poorly understood. Most randomized clinical trials have excluded patients with advanced liver disease, leaving limited evidence on the safety and efficacy of SGLT2is in this high-risk population.^4^ In fact, few large-scale studies with long-term follow-up have specifically examined the effects of any antidiabetic therapy in patients with coexisting T2D and cirrhosis, making the broader association of diabetes medications with cirrhotic outcomes largely unknown.^2^

T2D is a key factor underlying nonalcoholic fatty liver disease (NAFLD), promoting hepatic steatosis, inflammation, and fibrosis.^5^ Emerging evidence suggests that SGLT2is may confer hepatic benefits, including reductions in NAFLD incidence, cirrhosis progression, and composite liver-related outcomes, outperforming other glucose-lowering agents.^6^ A recent US nationwide cohort study^7^ found that SGLT2i use was also associated with lower risks of both hepatic and extra-hepatic complications—such as cardiovascular disease (CVD) and chronic kidney disease (CKD)—in patients with diabetes and metabolic dysfunction–associated steatotic liver disease; however, that study excluded individuals with viral hepatitis and did not specifically evaluate those with liver cirrhosis.

To address this knowledge gap, we evaluated the associations of SGLT2is with cirrhosis of all causes. Given the historically high prevalence of viral hepatitis in Taiwan,^8,9^ understanding their role in viral-related cirrhosis is of particular importance. We conducted a nationwide cohort study using Taiwan’s National Health Insurance Research Database (NHIRD) to compare the kidney, cardiovascular, and hepatic outcomes between SGLT2i and dipeptidyl peptidase–4 inhibitor (DPP4i) users with T2D and cirrhosis.

## Methods

### Data Source

This cohort study utilized the Taiwan NHIRD, established in 1995, which contains comprehensive medical records for approximately 23 million citizens. The NHI program has mandatory enrollment and captures outpatient visits, hospitalizations, procedures, and prescription records. Taiwan’s National Health Research Institute authorizes database access for scientific investigations without requiring individual patient consent. The study received approval from China Medical University Hospital’s research ethics committee and adhered to their established guidelines and regulations (CRREC-109-018). Our methodological approach followed the Strengthening the Reporting of Observational Studies in Epidemiology (STROBE) reporting guideline for cohort studies.

### Study Population and Design

We conducted a nationwide retrospective cohort analysis using deidentified data from Taiwan’s National Health Research Institutes. For privacy protection and to facilitate longitudinal tracking, personal identifiers were converted to unique encrypted codes. The study included adult patients with both T2D and liver cirrhosis diagnosed according to the *International Classification of Diseases, Ninth Revision, Clinical Modification (ICD-9-CM)* and *International Statistical Classification of Diseases, Tenth Revision, Clinical Modification (ICD-10-CM) *(detailed coding available in the eTable 1 in Supplement 1).

T2D was defined as at least 3 outpatient consultations or 1 hospital admission within 12 months, a definition previously validated using the NHIRD.^10^ We identified liver cirrhosis cases using a validated approach requiring either 2 sequential outpatient diagnoses or a single inpatient diagnosis—a method used in multiple studies.^11,12^ This diagnostic algorithm has demonstrated a positive predictive value of 82.6% when 2 diagnostic codes are present,^13^ and similar algorithms have demonstrated positive predictive values up to 98% in Taiwan.^14^ In our study, 89.2% of patients also underwent abdominal ultrasonography before the diagnosis, providing further support for case validity.

We identified 38 743 patients who initiated SGLT2is or DPP4is between May 1, 2016 (when SGLT2is were first approved in Taiwan), and December 31, 2023. The index date was defined as the first prescription date after both T2D and cirrhosis had been established. Follow-up commenced immediately at the index date (*t* = 0) to eliminate immortal time bias, ensuring an uninterrupted exposure timeline for both groups (eFigure 1 in Supplement 1).

**Figure 1.**
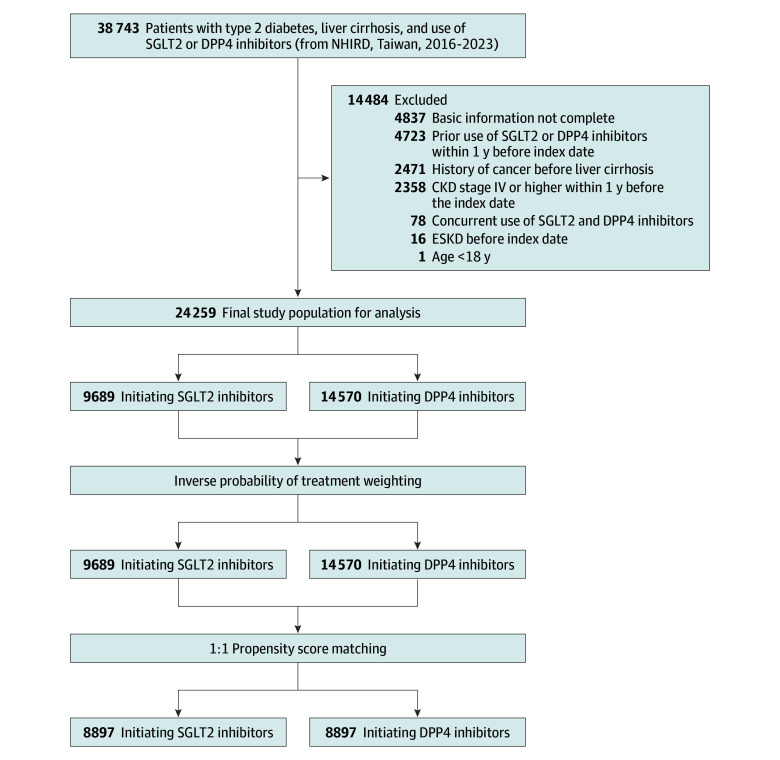
Participant Enrollment in Patients With Type 2 Diabetes and Liver Cirrhosis CKD indicates chronic kidney disease; DPP4, dipeptidyl peptidase–4; ESKD, end-stage kidney disease; NHIRD, National Health Insurance Research Database; SGLT2, sodium-glucose cotransporter–2

Exclusion criteria were age younger than 18 years, prior cancer, end-stage kidney disease (ESKD) or CKD stage IV or V before the index date, concurrent use of both medication classes, or any SGLT2i or DPP4i use within the preceding 12 months. Patients with cancer were excluded to focus on kidney and cardiovascular outcomes. The participant selection process is illustrated in Figure 1.

### Medication Exposure Assessment

This study examined 2 classes of antidiabetic medications: SGLT2is and DPP4is. SGLT2is included dapagliflozin, empagliflozin, and canagliflozin, while the DPP4is included alogliptin, linagliptin, sitagliptin, saxagliptin, and vildagliptin. We adopted an intention-to-treat approach to analyze data, evaluating outcomes on the basis of initial medication assignment regardless of subsequent treatment changes. To ensure clean exposure groups, we implemented a 365-day washout period (*t* = −365 to *t* = 0), excluding patients who had used either medication within 12 months before the index date.

### Outcome Measurements

The primary outcomes comprised ESKD, acute kidney injury (AKI), and major adverse cardiovascular events (MACE). ESKD cases were identified through the Taiwan Catastrophic Illness Registry, a system established for reimbursement purposes that ensures diagnostic accuracy. For AKI identification, we employed validated *ICD-9-CM* and *ICD-10-CM* codes with previously established diagnostic performance (positive predictive value, 98.5%; negative predictive value, 74.0%) in the NHIRD.^15^ MACE was defined as a composite end point encompassing acute myocardial infarction (AMI), stroke, heart failure (HF), and all-cause mortality. Secondary outcomes included individual components of MACE (hospitalization for AMI, stroke, and HF) and indicators of hepatic decompensation (hospitalization for hepatic encephalopathy, ascites, peritonitis, or esophageal variceal bleeding). Definitions for AMI, stroke, and HF have been previously validated in the NHIRD.^16,17^ Peritonitis (including spontaneous bacterial peritonitis and cirrhosis-related peritonitis) was identified using *ICD-9-CM* codes 567.2, 567.8, and 567.9 and *ICD-10-CM* code K65.2. In addition, to maximize diagnostic specificity, all primary outcomes were identified using inpatient diagnosis codes. Because inpatient-only definitions may preferentially capture more severe events, we additionally prespecified a sensitivity analysis incorporating outpatient diagnoses for AMI, stroke, and HF.

### Covariate Definition

Comorbidity profiles were assessed according to diagnostic codes documented within 1 year preceding the index date. The evaluated comorbidities included hypertension, hyperlipidemia, CVD, coronary artery disease, CKD, HF, viral hepatitis (both hepatitis B and C infections), nonalcoholic steatohepatitis, alcoholic cirrhosis, and hypoglycemia. Baseline cerebrovascular disease encompassed prior ischemic or hemorrhagic stroke, transient ischemic attack, and other chronic cerebrovascular disorders (*ICD-9-CM* codes 430-437 and *ICD-10-CM* codes I60-I63 and I66-I68), whereas the stroke MACE component referred to new-onset acute ischemic or hemorrhagic stroke during follow-up (*ICD-9-CM* codes 430-437, I61-I63, and I65-I66).

Medication use was assessed during the same 1-year baseline period. In addition to SGLT2is and DPP4is, we captured the use of glucagon-like peptide–1 receptor agonists, insulin, metformin, mineralocorticoid receptor antagonists, diuretics, statins, antiplatelet agents, renin-angiotensin system inhibitors (angiotensin-converting enzyme inhibitors and angiotensin II receptor blockers), carvedilol, and other nonselective β-blockers. For patients with viral hepatitis, we additionally captured the use of direct-acting antivirals, oral antiviral agents, and interferon-based therapies during the same period.

### Statistical Analysis

Descriptive statistics were reported as means (SDs) for continuous variables and frequencies (percentages) for categorical variables. To minimize confounding from baseline comorbidities and concomitant medication use, inverse probability of treatment weighting (IPTW) was employed. Propensity scores were estimated using multivariate logistic regression that included age, sex, index year, comorbidities, and medications use. Patients prescribed an SGLT2i received weights of 1 divided by the propensity score, and those prescribed a DPP4i received weights of 1 divided by (1 − propensity score). This method allocated greater analytical importance to individuals with lower probability of receiving their actual treatment. Stabilized weights were used to limit the impact of extreme values, as recommended in prior methodological work.^18^ The standardized mean difference, with a threshold of 0.10, was employed to evaluate the balance of covariates between groups.^19,20^

Patient follow-up commenced on the index date and continued until the occurrence of ESKD, AKI, MACE, hepatic decompensation, mortality, or the study termination (December 31, 2023), whichever came first. To account for competing mortality risks, we employed cause-specific hazard functions in all Cox regression analyses. Cumulative incidence functions accounting for the competing risk of death were estimated using the Aalen-Johansen estimator with IPTW. The Gray test was used to compare cumulative incidence between groups. Upon testing the proportional hazards assumption via Schoenfeld residual analysis and identifying violations, we implemented time-varying coefficient models in our weighted Cox proportional hazards regression. This approach allowed us to calculate both unadjusted and multivariable-adjusted hazard ratios (HRs) with corresponding 95% CIs for the association of SGLT2is with the risk of ESKD, AKI, MACE, and hepatic complications. To evaluate variations in treatment effects, we conducted additional stratified analyses examining the association of specific SGLT2i agents (dapagliflozin, empagliflozin, and canagliflozin) with our primary outcomes. We also performed subgroup analyses using adjusted Cox models to assess potential effect size modification by baseline comorbidities and concomitant medications.

Additionally, we conducted sensitivity analyses using propensity score trimming at various thresholds, 1:1 propensity score matching with a caliper width of 0.2 SDs,^21^ incorporation of outpatient diagnoses for AMI, stroke, and HF and stratified analyses by baseline CKD stage (defined by *ICD-9-CM* and *ICD-10-CM* codes) to assess the robustness of our findings. All statistical analyses were conducted using SAS version 9.4 (SAS Institute Inc) and Python version 3.11.7 (Python Software Foundation), with 2-sided *P* < .05 considered statistically significant.

## Results

### Baseline Characteristics

A total of 24 259 patients with both T2D and liver cirrhosis (mean [SD] age, 64.68 [11.95] years; 8229 female [33.92%]) were identified, including 9689 patients (39.94%) receiving SGLT2is and 14 570 patients (60.06%) receiving DPP4is. Before weighting, SGLT2i users were younger (mean [SD] age, 62.25 [11.16] vs 66.29 [12.18] years) and included fewer females (2709 patients [27.96%] vs 5520 patients [37.89%]) compared with DPP4i users (Table 1). Among all participants, 5007 (20.64) had hepatitis B virus infection, 3664 (15.10%) had hepatitis C virus infection, and 1636 (6.74%) had alcohol-related liver disease. Hyperlipidemia was more prevalent in SGLT2i than DPP4i users (5435 patients [56.09%] vs 6614 patients [45.39%]), whereas cerebrovascular disease, hepatitis C infection, stroke, and hypoglycemia were more common in the DPP4i group. Insulin (1312 patients [13.54%] vs 1249 patients [9.26%]) and statin use (3206 participants [33.09%] vs 3442 participants [23.62%]) were more common among SGLT2i than DPP4i users. SGLT2i users had longer diabetes duration than DPP4i users (mean [SD], 10.18 [3.29] vs 9.86 [6.34] years). After IPTW, all baseline characteristics were well-balanced between the 2 groups, with standardized mean differences less than 0.10.

**Table 1.** Baseline Characteristics After Inverse Probability of Treatment Weighting in Patients With Type 2 Diabetes Receiving DPP4 or SGLT2 Inhibitors

Characteristic	Patients, No. (%)			SMD	
	Overall (N = 24 259)	DPP4 inhibitors (n = 14 570)	SGLT2 inhibitors (n = 9689)	Unweighted	Weighted
Diabetes duration, mean (SD), y	9.99 (6.37)	9.86 (6.34)	10.18 (6.42)	0.05	0.00
Index year of receiving SGLT2 or DPP4 inhibitors					
2016	1825 (7.52)	1506 (10.34)	319 (3.29)	0.28	0.02
2017	2607 (10.75)	2019 (13.86)	588 (6.07)	0.26	0.01
2018	2688 (11.08)	1965 (13.49)	723 (7.46)	0.20	−0.01
2019	3059 (12.61)	1966 (13.49)	1093 (11.28)	0.07	−0.01
2020	3568 (14.71)	2085 (14.31)	1483 (15.31)	−0.03	0.00
2021	3751 (15.46)	1972 (13.53)	1779 (18.36)	−0.13	0.00
2022	3537 (14.58)	1664 (11.42)	1873 (19.33)	−0.22	0.00
2023	3224 (13.29)	1393 (9.56)	1831 (18.90)	−0.27	0.00
Age, y					
Mean (SD)	64.68 (11.95)	66.29 (12.18)	62.25 (11.16)	−0.35	−0.02
18-24	7 (0.03)	3 (0.02)	4 (0.04)	−0.01	0.00
25-34	111 (0.46)	58 (0.40)	53 (0.55)	−0.02	0.00
35-44	1060 (4.37)	513 (3.52)	547 (5.65)	−0.10	0.00
45-54	3731 (15.38)	1975 (13.56)	1756 (18.12)	−0.13	0.00
55-64	6976 (28.76)	3836 (26.33)	3140 (32.41)	−0.13	0.00
65-74	7224 (29.78)	4325 (29.68)	2899 (29.92)	−0.01	−0.01
75-84	3981 (16.41)	2886 (19.81)	1095 (11.30)	0.24	0.01
≥85	1169 (4.82)	974 (6.68)	195 (2.01)	0.23	0.03
Sex					
Female	8229 (33.92)	5520 (37.89)	2709 (27.96)	0.21	0.00
Male	16 030 (66.08)	9050 (62.11)	6980 (72.04)		
Comorbidity					
Hypertension	15 053 (62.05)	9045 (62.08)	6008 (62.01)	0.00	0.00
Hyperlipidemia	12 049 (49.67)	6614 (45.39)	5435 (56.09)	−0.22	0.00
Cerebral vascular disease	2276 (9.38)	1593 (10.93)	683 (7.05)	0.14	−0.01
Coronary artery disease	4101 (16.91)	2241 (15.38)	1860 (19.20)	−0.10	0.00
Chronic kidney disease	4811 (19.83)	2982 (20.47)	1829 (18.88)	0.04	0.02
Hepatitis B virus	5007 (20.64)	2924 (20.07)	2083 (21.50)	−0.04	0.00
Hepatitis C virus	3664 (15.10)	2447 (16.79)	1217 (12.56)	0.12	0.02
Acute myocardial infarction	392 (1.62)	175 (1.20)	217 (2.24)	−0.08	−0.01
Stroke	1786 (7.36)	1275 (8.75)	511 (5.27)	0.14	−0.01
Heart failure	1595 (6.57)	878 (6.03)	717 (7.40)	−0.05	0.01
Nonalcoholic steatohepatitis	281 (1.16)	142 (0.97)	139 (1.43)	−0.04	0.00
Alcoholic liver cirrhosis	1636 (6.74)	1087 (7.46)	549 (5.67)	0.07	0.00
Hypoglycemia	451 (1.86)	352 (2.42)	99 (1.02)	0.11	0.00
Medication (diabetes)					
GLP-1 RAs	138 (0.57)	43 (0.30)	95 (0.98)	−0.09	0.01
Insulin	2661 (10.97)	1349 (9.26)	1312 (13.54)	−0.14	−0.01
Metformin	7367 (30.37)	4435 (30.44)	2932 (30.26)	0.00	−0.02
Medication (other)					
Mineralocorticoid receptor antagonists	1504 (6.20)	1030 (7.07)	474 (4.89)	0.09	0.00
Other diuretics	2834 (11.68)	1887 (12.95)	947 (9.77)	0.10	0.00
Statins	6648 (27.40)	3442 (23.62)	3206 (33.09)	−0.21	−0.01
Antiplatelet agents	3672 (15.14)	2037 (13.98)	1635 (16.87)	−0.08	0.00
ACEIs and ARBs	8676 (35.76)	4931 (33.84)	3745 (38.65)	−0.10	0.00
Direct-acting antivirals	31 (0.13)	24 (0.16)	7 (0.07)	0.03	0.00
Oral antiviral agents	2365 (9.75)	1335 (9.16)	1030 (10.63)	−0.05	0.00
Interferon	6 (0.02)	6 (0.04)	0	NA	NA
Carvedilol	744 (3.07)	437 (3.00)	307 (3.17)	−0.01	−0.01
Other nonselective β-blockers	1738 (7.16)	1116 (7.66)	622 (6.42)	0.05	0.00

Abbreviations: ACEIs, angiotensin-converting enzyme inhibitors; ARBs, angiotensin receptor blockers; DPP4, dipeptidyl peptidase–4; GLP-1 RAs, glucagon-like peptide-1 receptor agonists; NA, not applicable; SGLT2, sodium-glucose cotransporter–2; SMD, standardized mean difference.

### SGLT2i Use and Clinical Outcomes

**Figure 2.**
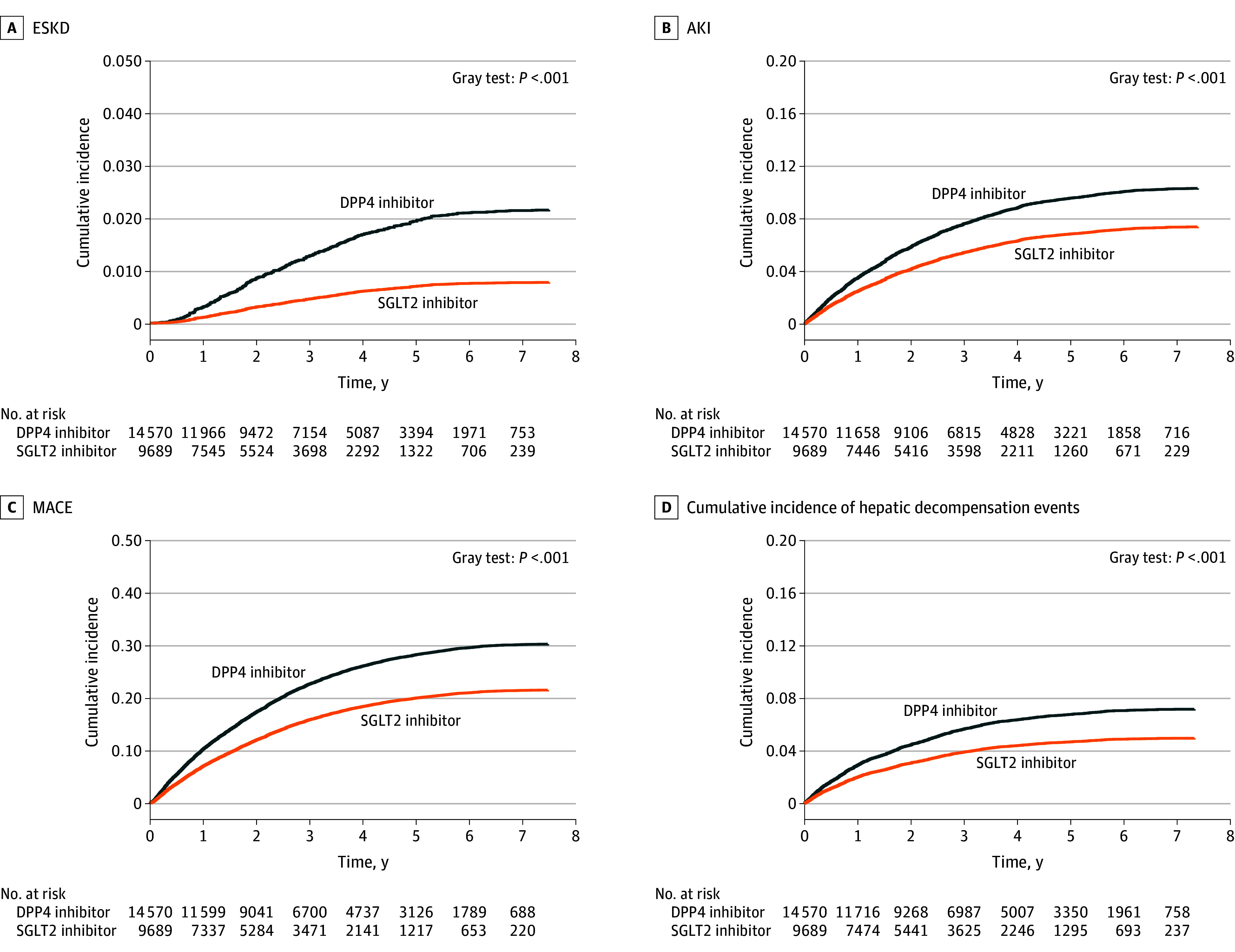
Cumulative Incidence Curves Comparing Outcomes for Sodium-Glucose Cotransporter–2 (SGLT2) vs Dipeptidyl Peptidase–4 (DPP4) Inhibitors in Patients With Type 2 Diabetes With Liver Cirrhosis A, Cumulative incidence of end-stage kidney disease (ESKD). B, Risk of acute kidney injury (AKI). C, Probability of major adverse cardiovascular events (MACE) in the inverse probability treatment-weighted population. D, Cumulative incidence of hepatic decompensation.

During a median (IQR) follow-up of approximately 2.3 (1.0-4.0) years, the cumulative incidence curves demonstrated a consistently lower probability of ESKD, AKI, and MACE in SGLT2i users compared with DPP4i users (Gray test *P* < .001) (Figure 2). In the weighted population, SGLT2i use was associated with significantly reduced risk of ESKD (adjusted HR, 0.34; 95% CI, 0.25-0.47; *P* < .001), AKI (adjusted HR, 0.66; 95% CI, 0.59-0.74; *P* < .001), and MACE (adjusted HR, 0.67; 95% CI, 0.62-0.71; *P* < .001) compared with DPP4i use after adjusting for diabetes duration, age, sex, comorbidities, and concomitant medications (Table 2). These associations remained stable under various propensity score trimming analyses (eTable 2 in Supplement 1).

**Table 2.** Adjusted Risk of Adverse Kidney and Cardiovascular Outcomes Among Patients With Type 2 Diabetes and Liver Cirrhosis Using SGLT2is or DPP4is

Exposure	Events, No.	Person-years	Incidence rate^a^	Crude HR (95% CI)	*P* value	Model 1, HR (95% CI)^b^	*P* value	Model 2, HR (95% CI)^c^	*P* value
End-stage kidney disease									
DPP4i (n = 14 570)	339	46 949.41	7.22	1 [Reference]	NA	1 [Reference]	NA	1 [Reference]	NA
SGLT2i									
Any (n = 9689)	60	26 096.79	2.30	0.33 (0.24-0.45)	<.001	0.33 (0.24-0.45)	<.001	0.34 (0.25-0.47)	<.001
Canagliflozin (n = 735)	4	1781.19	2.25	0.29 (0.11-0.79)	.02	0.28 (0.10-0.75)	.01	0.28 (0.10-0.75)	.01
Dapagliflozin (n = 4907)	26	12 983.44	2.00	0.31 (0.19-0.49)	<.001	0.31 (0.19-0.50)	<.001	0.33 (0.21-0.53)	<.001
Empagliflozin (n = 4047)	30	11 332.16	2.65	0.36 (0.24-0.54)	<.001	0.35 (0.23-0.53)	<.001	0.37 (0.24-0.56)	<.001
Acute kidney injury									
DPP4i (n = 14 570)	1708	45 291.06	37.71	1 [Reference]	NA	1 [Reference]	NA	1 [Reference]	NA
SGLT2i									
Any (n = 9689)	568	25 573.71	22.21	0.65 (0.58-0.73)	<.001	0.66 (0.59-0.73)	<.001	0.66 (0.59-0.74)	<.001
Canagliflozin (n = 735)	37	1763.15	20.99	0.65 (0.45-0.94)	.02	0.63 (0.44-0.91)	.014	0.63 (0.44-0.91)	.01
Dapagliflozin (n = 4907)	268	12 736.44	21.04	0.60 (0.52-0.70)	<.001	0.61 (0.52-0.70)	<.001	0.62 (0.54-0.73)	<.001
Empagliflozin (n = 4047)	263	11 074.11	23.75	0.72 (0.61-0.84)	<.001	0.72 (0.61-0.84)	<.001	0.69 (0.59-0.82)	<.001
MACE									
DPP4i (n = 14 570)	5132	44 740.75	114.71	1 [Reference]	NA	1 [Reference]	NA	1 [Reference]	NA
SGLT2i									
Any (n = 9689)	1640	25 061.92	65.44	0.66 (0.61-0.70)	<.001	0.67 (0.63-0.72)	<.001	0.67 (0.62-0.71)	<.001
Canagliflozin (n = 735)	116	1728.80	67.10	0.71 (0.58-0.88)	.002	0.70 (0.56-0.86)	.001	0.71 (0.58-0.88)	.001
Dapagliflozin (n = 4907)	811	12 458.70	65.10	0.66 (0.60-0.72)	<.001	0.68 (0.62-0.74)	<.001	0.69 (0.63-0.76)	<.001
Empagliflozin (n = 4047)	713	10 874.41	65.57	0.65 (0.59-0.71)	<.001	0.66 (0.60-0.72)	<.001	0.63 (0.58-0.69)	<.001

Abbreviations: DPP-4i, dipeptidyl peptidase–4 inhibitor; HR, hazard ratio; MACE, major adverse cardiovascular events; NA, not applicable; SGLT2i, sodium-glucose cotransporter–2 inhibitor.

^a^ Incidence rate is equal to the number of events per 1000 person-years.

^b^ Model 1 adjusted for diabetes duration, age, and sex.

^c^ Model 2 adjusted for diabetes duration, age, sex, comorbidities, and medications.

Subgroup analyses showed broadly consistent findings across categories defined by age, sex, comorbidities, and medication use (eTable 3 in Supplement 1). Some smaller subgroups, such as those with prior AMI or hypoglycemia, had wider 95% CIs, but effect size estimates remained directionally similar.

**Figure 3.**
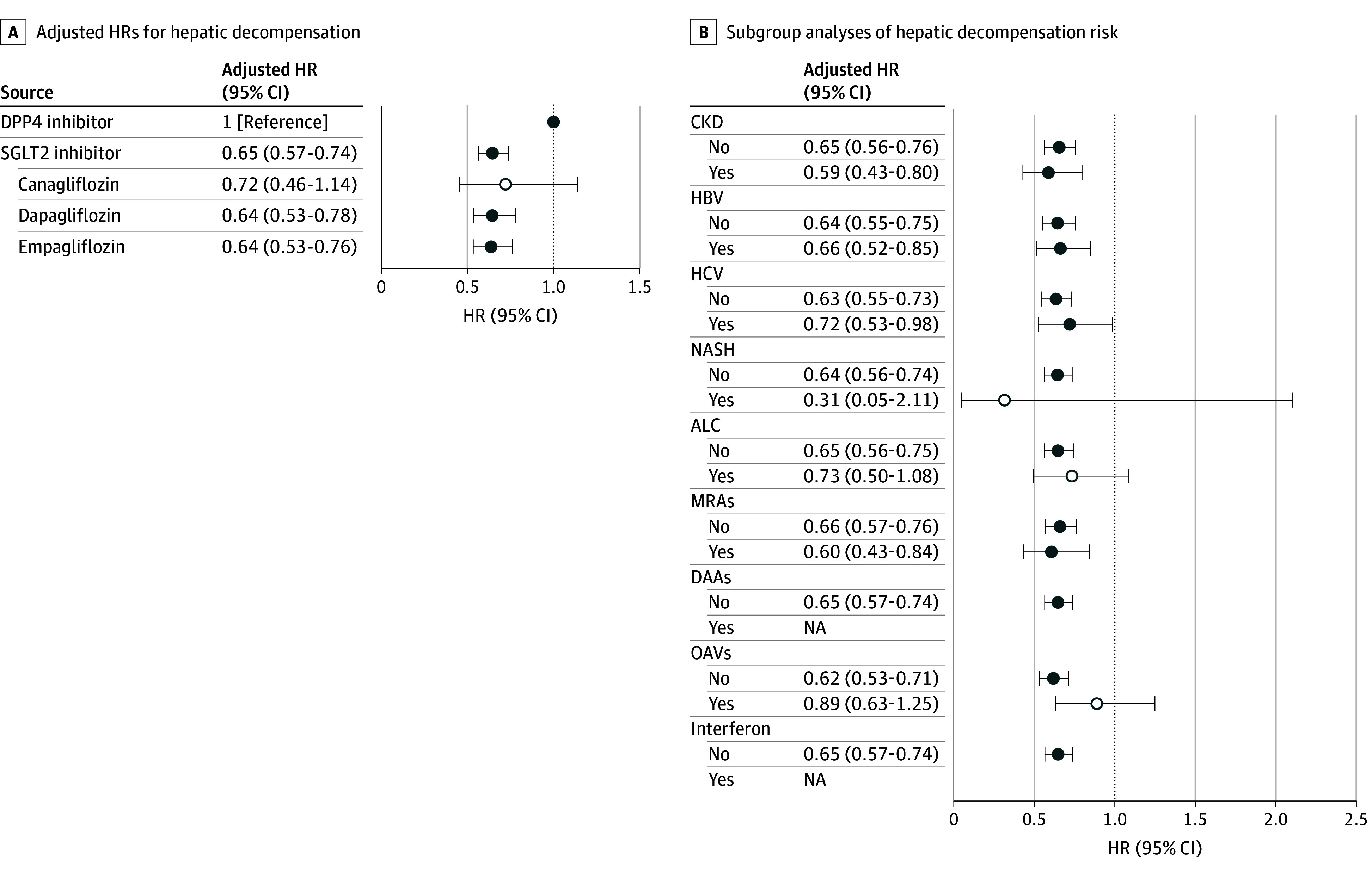
Forest Plots for Hazard of Hepatic Decompensation Risk Comparing Sodium-Glucose Cotransporter–2 (SGLT2) vs Dipeptidyl Peptidase–4 (DPP4) Inhibitors in Patients With Type 2 Diabetes With Liver Cirrhosis A, Adjusted hazard ratios (HRs) for hepatic decompensation by SGLT2 inhibitor type. B, Subgroup analyses of associations of treatment with hepatic decompensation risk stratified by comorbidities and concomitant medications. Solid circles denote estimates that are statistically significant (*P* < .05), where the 95% CIs do not cross the null value (HR = 1.00). Open circles denote estimates that did not reach statistical significance (*P* ≥ .05). ALC indicates alcoholic cirrhosis; CKD, chronic kidney disease; DAAs, direct-acting antivirals; HBV, hepatitis B virus; HCV, hepatitis C virus; MRAs, mineralocorticoid receptor antagonists; NA, not applicable; NASH, nonalcoholic steatohepatitis; OAVs, oral antiviral agents.

For hepatic decompensation, SGLT2i therapy was associated with a lower incidence compared with DPP4i therapy (Gray test *P* < .001) (Figure 3A), even adjusting for other risk factors (HR, 0.65; 95% CI, 0.57-0.74; *P* < .001) (Figure 3B). In the subgroup analysis, the beneficial association of SGLT2is was consistent across most key subgroups (Figure 3C), including patients with and without CKD or hepatitis B infection. Although estimates also favored SGLT2is in smaller subgroups, such as those with hepatitis C virus or nonalcoholic steatohepatitis, 95% CIs were wider and did not reach statistical significance.

For MACE components, SGLT2i users exhibited significantly lower risks of AMI (adjusted HR, 0.60; 95% CI, 0.45-0.80; *P* < .001), stroke (adjusted HR, 0.72; 95% CI, 0.61-0.85; *P* < .001), and all-cause mortality (adjusted HR, 0.58; 95% CI, 0.53-0.63; *P* < .001), whereas there was no significant association with HF (eFigure 2 in Supplement 1). Additionally, SGLT2i use was associated with reduced risk of hepatic decompensation events, particularly ascites (adjusted HR, 0.65; 95% CI, 0.55-0.76; *P* < .001), peritonitis (adjusted HR, 0.62; 95% CI, 0.45-0.86; *P* < .001), and esophageal variceal bleeding (adjusted HR, 0.67; 95% CI, 0.45-0.99; *P* = .04).

### Sensitivity Analysis

We included 17 794 patients (8897 in each group) in the propensity score–matched cohort. Baseline characteristics were well-balanced between groups (all standardized mean differences <0.10) (eTable 4 in Supplement 1). In the matched cohort, SGLT2i use remained associated with significantly reduced risks of ESKD (adjusted HR, 0.30; 95% CI, 0.15-0.60; *P* < .001), AKI (adjusted HR, 0.66; 95% CI, 0.56-0.78; *P* < .001), MACE (adjusted HR, 0.63; 95% CI, 0.58-0.69; *P* < .001), and hepatic decompensation (adjusted HR, 0.63; 95% CI, 0.51-0.78; *P* < .001) (eTable 5 in Supplement 1), findings consistent with our primary IPTW analysis.

In addition, sensitivity analyses incorporating outpatient diagnoses of AMI, stroke, and HF showed similar findings (eTable 6 in Supplement 1). In stratified analyses by CKD stage, the protective associations of SGLT2is with ESKD, AKI, MACE, and hepatic decompensation remained evident regardless of baseline kidney function (eTable 7 in Supplement 1).

## Discussion

This cohort study provides new evidence supporting the use of SGLT2is in patients with T2D and liver cirrhosis, demonstrating significant associations with reductions in the risks of ESKD, AKI, and MACE, as well as hepatic decompensation events. Importantly, these protective associations were consistent across the full spectrum of cirrhosis causes, including viral hepatitis, alcoholic liver disease, and nonalcoholic steatohepatitis, and remained robust after multivariable adjustment. These findings extend the established cardiorenal benefits of SGLT2is to a population previously underrepresented in clinical trials.

In our cohort, baseline characteristics indicated that about one-fifth of patients had hepatitis B virus infection, 15% had hepatitis C virus infection, and 7% had alcohol-related liver disease, suggesting viral hepatitis as the predominant underlying condition in this cirrhotic population. Because all patients had T2D, NAFLD was likely a major cause among the remaining cases. Given the shared metabolic pathways, we also referred to some NAFLD studies to extend our understanding of SGLT2i effects in patients with cirrhosis with metabolic risk factors.

CVD is the leading cause of death in patients with NAFLD,^22^ and NAFLD was associated with a 90% increased CVD risk in patients with T2D,^23^ due to poor glycemic control, insulin resistance, inflammation, and oxidative stress,^22^ all of which are improved by SGLT2is.^24^ Cirrhosis may further exacerbate cardiovascular dysfunction through hyperdynamic circulation, systemic inflammation, and neurohormonal dysregulation, resembling decompensated HF.^4^ SGLT2is have shown benefits in HF with both preserved and reduced ejection fraction,^25^ likely through natriuresis, hemodynamic relief, and anti-inflammatory effects, which may also explain the reduced MACE risk in patients with cirrhosis using SGLT2is.^4^ Our findings suggest that SGLT2is retain their cardioprotective effects in patients with T2D and liver cirrhosis.

NAFLD also increases the risk of CKD, through inflammation, and metabolic pathways,^26^ and greater NAFLD severity correlates with higher kidney risk.^27^ In parallel, AKI is a frequent and severe complication in cirrhosis,^28^ with kidney failure significantly increasing mortality by 7-fold.^29^ The risk of dialysis is highest in patients with both T2D and cirrhosis,^3^ underscoring the importance of preventing both CKD and AKI in this population.

SGLT2is have demonstrated protective kidney effects in CKD progression and reduced AKI risk in both clinical trials^30^ and cohort studies.^31^ In our study, these kidney benefits were preserved in patients with T2D and cirrhosis. One possible mechanism is the natriuretic effect of SGLT2is, which may reduce ascites formation and limit the need for traditional diuretics, particularly mineralocorticoid receptor antagonists and loop diuretics, which are often associated with hepatorenal syndrome. SGLT2i use has been shown to safely reduce ascites recurrence without compromising kidney function,^4^ and is associated with lower ascites-related mortality compared with DPP4is.^32^ These findings suggest SGLT2is may mitigate kidney complications in this high-risk population by reducing diuretic-associated kidney injury.

We also observed a reduction in hepatic decompensation events. While the reduction in peritonitis risk is a novel observation, it is supported by a prior meta-analysis showing decreased rates of gastroenteritis with SGLT2i use.^33^ Moreover, SGLT2is have been associated with reduced esophageal variceal bleeding risk compared with DPP4is^34^ and thiazolidinedione,^35^ supporting the hepatic benefits observed in our cohort.

Viral hepatitis has been associated with worse cardiovascular^36^ and kidney outcomes,^37^ potentially through mechanisms such as atherogenesis, proinflammatory cytokines, and oxidative stress.^38^ Steatosis in chronic hepatitis B virus infection may further contribute to lipid peroxidation and systemic inflammation,^39^ suggesting a potential therapeutic role for SGLT2is in this setting. A recent study reported reduced hepatocellular carcinoma risk in patients with T2D and hepatitis B treated with SGLT2is.^40^ Although data remain limited, our results suggest that the cardiorenal benefits of SGLT2is appear to extend to viral hepatitis-related cirrhosis as well.

### Strengths and Limitations

Strengths of this study include the use of a large, nationwide cohort, validated diagnostic algorithms, and comprehensive adjustment for comorbidities and medication use. IPTW enhanced covariate balance while retaining statistical power. Our study addresses potential lead-time bias through several design features. First, we adjusted for both diabetes duration and index year in our propensity score model to account for temporal changes in prescribing practices. Second, after IPTW, SGLT2i users had longer diabetes duration than DPP4i users (10.18 vs 9.86 years), confirming that treatment was not initiated earlier in the disease course.

Some limitations should be acknowledged. The lack of laboratory data precluded assessment of cirrhosis severity, and lifestyle factors such as alcohol consumption were unavailable. Although alcoholic cirrhosis was included as a diagnostic category, residual confounding cannot be fully excluded. Furthermore, despite the use of IPTW to balance baseline characteristics between SGLT2i and DPP4i users, confounding by indication may still persist.

## Conclusions

In this cohort study, we found that SGLT2is were associated with significantly lower risk of ESKD, AKI, MACE, and hepatic decompensation among patients with T2D and cirrhosis. By interrupting key metabolic and hemodynamic pathways underlying T2D and liver disease, SGLT2is may offer meaningful clinical benefits for this high-risk population. Prospective studies are warranted to confirm these findings.

## Notes

Supplement 1. eFigure 1. Timeline of Our Study eTable 1. Classification of Diseases and Medications Under Study Based on the International Statistical Classification of Diseases (ICD-9 and ICD-10) and the Anatomical Therapeutic Chemical (ATC) System eTable 2. Sensitivity Analysis of Adverse Outcomes Using Various Propensity Score Trimming Thresholds (IPTW Analysis) eTable 3. Subgroup Hazard Ratios for ESRD, AKI, and MACE Associated With SGLT2 vs. DPP4 Inhibitors (IPTW Analysis) eFigure 2. Hazard Ratios for MACE and Hepatic Decompensation: SGLT2i vs DPP4i in T2DM Patients With Cirrhosis eTable 4. Baseline Characteristics After Propensity Score Matching in Patients With Type 2 Diabetes Receiving DPP4 or SGLT2 Inhibitors eTable 5. Adjusted Risk of Adverse Renal and Cardiovascular Outcomes Among Patients With Type 2 Diabetes and Liver Cirrhosis Using SGLT2 or DPP4 Inhibitors (propensity score matching) eTable 6. Hazard Ratios for AMI, Stroke, and HF Associated with SGLT2 vs. DPP4 Inhibitors by Visit Type (IPTW Analysis) eTable 7. Hazard Ratios for ESRD, AKI, and MACE Associated With SGLT2 vs. DPP4 Inhibitors by CKD Stage (IPTW Analysis)

Supplement 2. Data Sharing Statement

## References

[1] Lee WG, Wells CI, McCall JL, Murphy R, Plank LD. Prevalence of diabetes in liver cirrhosis: a systematic review and meta-analysis. Diabetes Metab Res Rev. 2019;35(6):e3157. doi:10.1002/dmrr.3157 30901133

[2] Elkrief L, Rautou PE, Sarin S, Valla D, Paradis V, Moreau R. Diabetes mellitus in patients with cirrhosis: clinical implications and management. Liver Int. 2016;36(7):936-948. doi:10.1111/liv.13115 26972930

[3] Sheen YJ, Kung PT, Sheu WH, Kuo WY, Tsai WC. Impact of liver cirrhosis on incidence of dialysis among patients with type 2 diabetes. Diabetes Ther. 2020;11(11):2611-2628. doi:10.1007/s13300-020-00919-6 32901421 PMC7547941

[4] Siafarikas C, Kapelios CJ, Papatheodoridi M, Vlachogiannakos J, Tentolouris N, Papatheodoridis G. Sodium-glucose linked transporter 2 inhibitors in liver cirrhosis: beyond their antidiabetic use. Liver Int. 2024;44(4):884-893. doi:10.1111/liv.15851 38293770

[5] Hickman IJ, Macdonald GA. Impact of diabetes on the severity of liver disease. Am J Med. 2007;120(10):829-834. doi:10.1016/j.amjmed.2007.03.025 17904449

[6] Khanmohammadi S, Habibzadeh A, Kamrul-Hasan ABM, Schuermans A, Kuchay MS. Glucose-lowering drugs and liver-related outcomes among individuals with type 2 diabetes: a systematic review of longitudinal population-based studies. Diabet Med. 2024;41(11):e15437. doi:10.1111/dme.15437 39340770

[7] Mao X, Zhang X, Kam L, . Synergistic association of sodium-glucose cotransporter-2 inhibitor and metformin on liver and non-liver complications in patients with type 2 diabetes mellitus and metabolic dysfunction-associated steatotic liver disease. Gut. 2024;73(12):2054-2061. doi:10.1136/gutjnl-2024-332481 39122360

[8] Yu ML, Chen PJ, Dai CY, . 2020 Taiwan consensus statement on the management of hepatitis C: part (I) general population. J Formos Med Assoc. 2020;119(6):1019-1040. doi:10.1016/j.jfma.2020.04.003 32359879

[9] Liu CJ, Chen PJ. Elimination of hepatitis B in highly endemic settings: lessons learned in Taiwan and challenges ahead. Viruses. 2020;12(8):815. doi:10.3390/v12080815 32731536 PMC7472725

[10] Lin CC, Lai MS, Syu CY, Chang SC, Tseng FY. Accuracy of diabetes diagnosis in health insurance claims data in Taiwan. J Formos Med Assoc. 2005;104(3):157-163. 15818428

[11] Wu VC, Chen SW, Ting PC, . Selection of β-blocker in patients with cirrhosis and acute myocardial infarction: a 13-year nationwide population-based study in Asia. J Am Heart Assoc. 2018;7(19):e008982. doi:10.1161/JAHA.118.008982 30371327 PMC6404872

[12] Lin YT, Wu PH, Lin CY, . Cirrhosis as a risk factor for tuberculosis infection–a nationwide longitudinal study in Taiwan. Am J Epidemiol. 2014;180(1):103-110. doi:10.1093/aje/kwu095 24829509

[13] Nehra MS, Ma Y, Clark C, Amarasingham R, Rockey DC, Singal AG. Use of administrative claims data for identifying patients with cirrhosis. J Clin Gastroenterol. 2013;47(5):e50-e54. doi:10.1097/MCG.0b013e3182688d2f 23090041 PMC3556340

[14] Ng KJ, Lee YK, Huang MY, Hsu CY, Su YC. Risks of venous thromboembolism in patients with liver cirrhosis: a nationwide cohort study in Taiwan. J Thromb Haemost. 2015;13(2):206-213. doi:10.1111/jth.12805 25471737

[15] Wu VC, Wu CH, Huang TM, ; NSARF Group. Long-term risk of coronary events after AKI. J Am Soc Nephrol. 2014;25(3):595-605. doi:10.1681/ASN.2013060610 24503241 PMC3935592

[16] Cheng CL, Lee CH, Chen PS, Li YH, Lin SJ, Yang YH. Validation of acute myocardial infarction cases in the national health insurance research database in Taiwan. J Epidemiol. 2014;24(6):500-507. doi:10.2188/jea.JE20140076 25174915 PMC4213225

[17] Hsieh CY, Chen CH, Li CY, Lai ML. Validating the diagnosis of acute ischemic stroke in a National Health Insurance claims database. J Formos Med Assoc. 2015;114(3):254-259. doi:10.1016/j.jfma.2013.09.009 24140108

[18] Chesnaye NC, Stel VS, Tripepi G, . An introduction to inverse probability of treatment weighting in observational research. Clin Kidney J. 2021;15(1):14-20. doi:10.1093/ckj/sfab158 35035932 PMC8757413

[19] Franklin JM, Rassen JA, Ackermann D, Bartels DB, Schneeweiss S. Metrics for covariate balance in cohort studies of causal effects. Stat Med. 2014;33(10):1685-1699. doi:10.1002/sim.6058 24323618

[20] Zhang Z, Kim HJ, Lonjon G, Zhu Y; AME Big-Data Clinical Trial Collaborative Group. Balance diagnostics after propensity score matching. Ann Transl Med. 2019;7(1):16. doi:10.21037/atm.2018.12.10 30788363 PMC6351359

[21] Austin PC. Optimal caliper widths for propensity-score matching when estimating differences in means and differences in proportions in observational studies. Pharm Stat. 2011;10(2):150-161. doi:10.1002/pst.433 20925139 PMC3120982

[22] Targher G, Lonardo A, Byrne CD. Nonalcoholic fatty liver disease and chronic vascular complications of diabetes mellitus. Nat Rev Endocrinol. 2018;14(2):99-114. doi:10.1038/nrendo.2017.173 29286050

[23] Targher G, Bertolini L, Rodella S, . Nonalcoholic fatty liver disease is independently associated with an increased incidence of cardiovascular events in type 2 diabetic patients. Diabetes Care. 2007;30(8):2119-2121. doi:10.2337/dc07-0349 17519430

[24] Makri ES, Goulas A, Polyzos SA. Sodium-glucose co-transporter 2 inhibitors in nonalcoholic fatty liver disease. Eur J Pharmacol. 2021;907:174272. doi:10.1016/j.ejphar.2021.174272 34147478

[25] Talha KM, Anker SD, Butler J. SGLT-2 Inhibitors in heart failure: a review of current evidence. Int J Heart Fail. 2023;5(2):82-90. doi:10.36628/ijhf.2022.0030 37180562 PMC10172076

[26] Targher G, Chonchol M, Zoppini G, Abaterusso C, Bonora E. Risk of chronic kidney disease in patients with non-alcoholic fatty liver disease: is there a link? J Hepatol. 2011;54(5):1020-1029. doi:10.1016/j.jhep.2010.11.007 21145850

[27] Mantovani A, Zaza G, Byrne CD, . Nonalcoholic fatty liver disease increases risk of incident chronic kidney disease: a systematic review and meta-analysis. Metabolism. 2018;79:64-76. doi:10.1016/j.metabol.2017.11.003 29137912

[28] Garcia-Tsao G, Parikh CR, Viola A. Acute kidney injury in cirrhosis. Hepatology. 2008;48(6):2064-2077. doi:10.1002/hep.22605 19003880

[29] Fede G, D’Amico G, Arvaniti V, . Renal failure and cirrhosis: a systematic review of mortality and prognosis. J Hepatol. 2012;56(4):810-818. doi:10.1016/j.jhep.2011.10.016 22173162

[30] Nuffield Department of Population Health Renal Studies Group; SGLT2 inhibitor Meta-Analysis Cardio-Renal Trialists’ Consortium. Impact of diabetes on the effects of sodium glucose co-transporter-2 inhibitors on kidney outcomes: collaborative meta-analysis of large placebo-controlled trials. Lancet. 2022;400(10365):1788-1801. doi:10.1016/S0140-6736(22)02074-8 36351458 PMC7613836

[31] Chung MC, Hung PH, Hsiao PJ, . Sodium-glucose transport protein 2 inhibitor use for type 2 diabetes and the incidence of acute kidney injury in Taiwan. JAMA Netw Open. 2023;6(2):e230453. doi:10.1001/jamanetworkopen.2023.0453 36811856 PMC9947724

[32] Saffo S, Kaplan DE, Mahmud N, . Impact of SGLT2 inhibitors in comparison with DPP4 inhibitors on ascites and death in veterans with cirrhosis on metformin. Diabetes Obes Metab. 2021;23(10):2402-2408. doi:10.1111/dom.14488 34227216 PMC8429193

[33] Puckrin R, Saltiel MP, Reynier P, Azoulay L, Yu OHY, Filion KB. SGLT-2 inhibitors and the risk of infections: a systematic review and meta-analysis of randomized controlled trials. Acta Diabetol. 2018;55(5):503-514. doi:10.1007/s00592-018-1116-0 29484489

[34] Kawaguchi T, Fujishima Y, Wakasugi D, . Effects of SGLT2 inhibitors on the onset of esophageal varices and extrahepatic cancer in type 2 diabetic patients with suspected MASLD: a nationwide database study in Japan. J Gastroenterol. 2024;59(12):1120-1132. doi:10.1007/s00535-024-02158-z 39392481 PMC11541318

[35] Bea S, Ko HY, Bae JH, . Risk of hepatic events associated with use of sodium-glucose cotransporter-2 inhibitors versus glucagon-like peptide-1 receptor agonists, and thiazolidinediones among patients with metabolic dysfunction-associated steatotic liver disease. Gut. 2025;74(2):284-294. doi:10.1136/gutjnl-2024-332687 39242193 PMC11874371

[36] Lee KK, Stelzle D, Bing R, . Global burden of atherosclerotic cardiovascular disease in people with hepatitis C virus infection: a systematic review, meta-analysis, and modelling study. Lancet Gastroenterol Hepatol. 2019;4(10):794-804. doi:10.1016/S2468-1253(19)30227-4 31377134 PMC6734111

[37] Fabrizi F, Donato FM, Messa P. Association between hepatitis B virus and chronic kidney disease: a systematic review and meta-analysis. Ann Hepatol. 2017;16(1):21-47. doi:10.5604/16652681.1226813 28051791

[38] Adinolfi LE, Zampino R, Restivo L, . Chronic hepatitis C virus infection and atherosclerosis: clinical impact and mechanisms. World J Gastroenterol. 2014;20(13):3410-3417. doi:10.3748/wjg.v20.i13.3410 24707124 PMC3974508

[39] Shi YW, Yang RX, Fan JG. Chronic hepatitis B infection with concomitant hepatic steatosis: current evidence and opinion. World J Gastroenterol. 2021;27(26):3971-3983. doi:10.3748/wjg.v27.i26.3971 34326608 PMC8311534

[40] Lee CH, Mak LY, Tang EH, . SGLT2i reduces risk of developing HCC in patients with co-existing type 2 diabetes and hepatitis B infection: a territory-wide cohort study in Hong Kong. Hepatology. 2023;78(5):1569-1580. doi:10.1097/HEP.0000000000000404 37055020

