# Supplementary material for: SGLT2 Inhibitor Use and Cardiorenal Outcomes in Type 2 Diabetes With Liver Cirrhosis
Source: JAMA Netw Open. 2026 Feb 23;9(2):e2560429. doi: 10.1001/jamanetworkopen.2025.60429 (PMC12930281; doi:10.1001/jamanetworkopen.2025.60429)
Supplement: Supplement 1. — eFigure 1. Timeline of Our Study eTable 1. Classification of Diseases and Medications Under Study Based on the International Statistical Classification of Diseases (ICD-9 and ICD-10) and the Anatomical Therapeutic Chemical (ATC) System eTable 2. Sensitivity Analysis of Adverse Outcomes Using Various Propensity Score Trimming Thresholds (IPTW Analysis) eTable 3. Subgroup Hazard Ratios for ESRD, AKI, and MACE Associated With SGLT2 vs. DPP4 Inhibitors (IPTW Analysis) eFigure 2. Hazard Ratios for MACE and Hepatic Decompensation: SGLT2i vs DPP4i in T2DM Patients With Cirrhosis eTable 4. Baseline Characteristics After Propensity Score Matching in Patients With Type 2 Diabetes Receiving DPP4 or SGLT2 Inhibitors eTable 5. Adjusted Risk of Adverse Renal and Cardiovascular Outcomes Among Patients With Type 2 Diabetes and Liver Cirrhosis Using SGLT2 or DPP4 Inhibitors (propensity score matching) eTable 6. Hazard Ratios for AMI, Stroke, and HF Associated with SGLT2 vs. DPP4 Inhibitors by Visit Type (IPTW Analysis) eTable 7. Hazard Ratios for ESRD, AKI, and MACE Associated With SGLT2 vs. DPP4 Inhibitors by CKD Stage (IPTW Analysis) [file jamanetwopen-e2560429-s001.pdf]

Supplemental Online Content

Chung M-C, Yu T-M, Wu L-Y, Wu M-J, Shieh J-J, Chung C-J. SGLT2 inhibitor use and cardiorenal outcomes in type 2 diabetes with liver cirrhosis. *JAMA Netw. Open*. 2026;9(2):e2560429. doi:10.1001/jamanetworkopen.2025.60429

eFigure 1. Timeline of Our Study

eTable 1. Classification of Diseases and Medications Under Study Based on the International Statistical Classification of Diseases (ICD-9 and ICD-10) and the Anatomical Therapeutic Chemical (ATC) System

eTable 2. Sensitivity Analysis of Adverse Outcomes Using Various Propensity Score Trimming Thresholds (IPTW Analysis)

eTable 3. Subgroup Hazard Ratios for ESRD, AKI, and MACE Associated With SGLT2 vs. DPP4 Inhibitors (IPTW Analysis)

eFigure 2. Hazard Ratios for MACE and Hepatic Decompensation: SGLT2i vs DPP4i in T2DM Patients With Cirrhosis

eTable 4. Baseline Characteristics After Propensity Score Matching in Patients With Type 2 Diabetes Receiving DPP4 or SGLT2 Inhibitors

eTable 5. Adjusted Risk of Adverse Renal and Cardiovascular Outcomes Among Patients With Type 2 Diabetes and Liver Cirrhosis Using SGLT2 or DPP4 Inhibitors (propensity score matching)

eTable 6. Hazard Ratios for AMI, Stroke, and HF Associated with SGLT2 vs. DPP4 Inhibitors by Visit Type (IPTW Analysis)

eTable 7. Hazard Ratios for ESRD, AKI, and MACE Associated With SGLT2 vs. DPP4 Inhibitors by CKD Stage (IPTW Analysis)

This supplemental material has been provided by the authors to give readers additional information about their work.

© 2026 Chung M-C et al. *JAMA Network Open*.

eFigure 1. Timeline of our study.

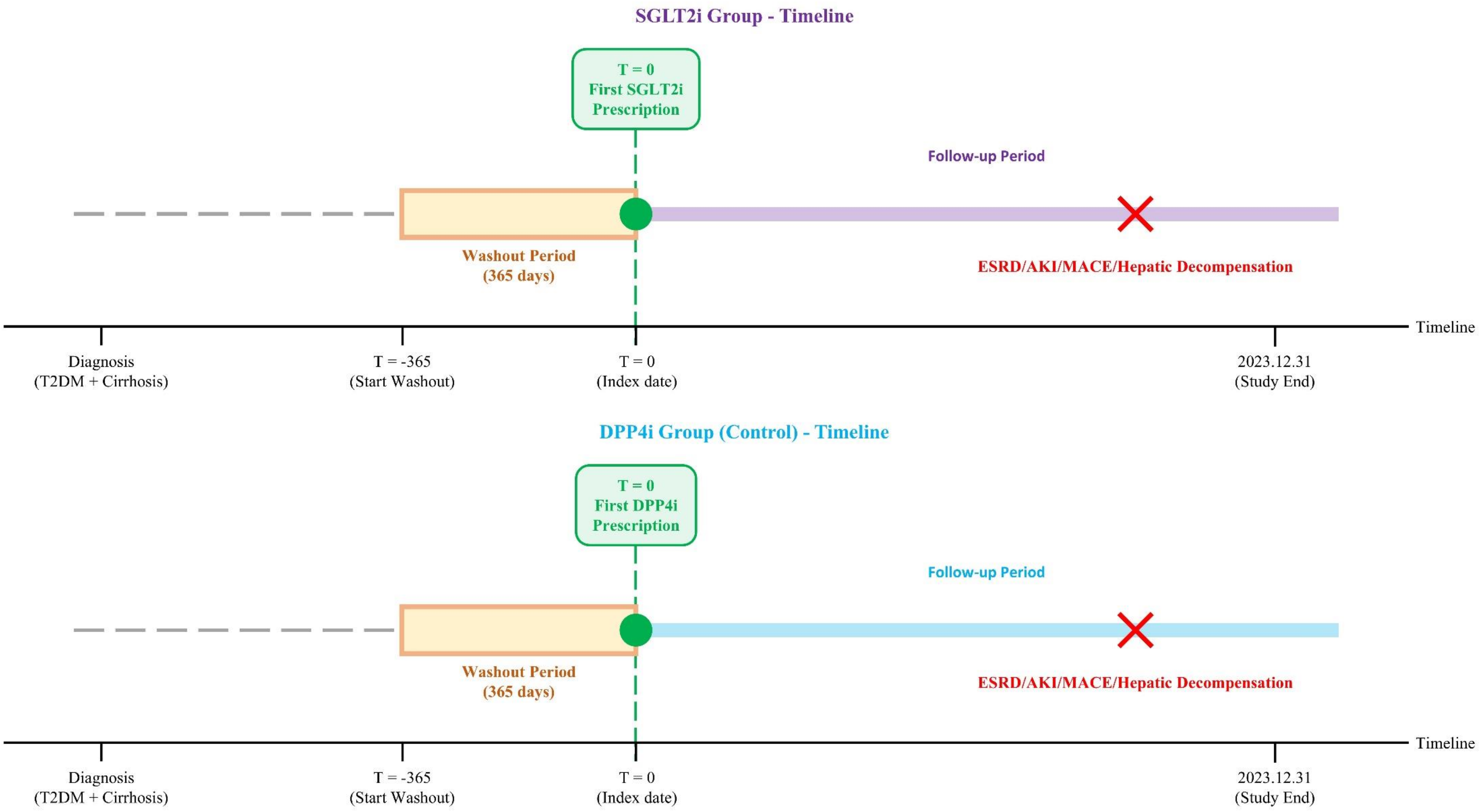

© 2026 Chung M-C et al. *JAMA Network Open*.

eTable 1. Classification of Diseases and Medications Under Study Based on the International Statistical Classification of Diseases (ICD-9 and ICD-10) and the Anatomical Therapeutic Chemical (ATC) System

Name	Code
Disease	
T2DM	250, E11
Cirrhosis	5712, 571.5, 571.6, K70.2, K70.3, K74.1 - K74.6
ESRD	585, N18
Cancer	140 - 208, C00 - C97
AKI	584, N17
ESRD	585, N18
MACE	410, 428, 430 - 437, I21, I22, I50, I61 - I63, I65, I66
AMI	410, I21, I22
Stroke	430 - 437, I61 - I63, I65, I66
HF	428, I50
Hepatic Decompensation	456.0, 456.20, 567.2, 567.8, 567.9, 572.2, 789.5, I85.01, K65.2, K76.82, R18.8
Hepatic Encephalopathy	572.2, K76.82
Ascites	789.5, R18.8
Peritonitis	567.2, 567.8, 567.9, K65.2
EV Bleeding	456.0, 456.20, I85.01
HTN	401 - 405, I10 - I13, I15, I16
HPL	272, E78
CVD	430 - 437, I60 - I63, I66 - I68
CAD	410 - 414, I20 - I25
CKD	585, 403.01, 403.11, 403.91, 249.40, N18, E08.22, E09.22, E10.22, E11.22, E13.22, I12.0, I12.9, I13.10, I13.11
HF	428, I50
HBV	070.22, 070.23, 070.20, B18.0, B18.1, B19.1
HCV	070.44, 070.54, 070.41, 070.51, B18.2, B19.2
NASH	K75.81
ALC	571.2, K70.30
Hypoglycemia	251.0, 251.1, 251.2, E15, E16.0, E16.1, E16.2
Medication	

© 2026 Chung M-C et al. *JAMA Network Open*.

DPP4 inhibitor	A10BH
SGLT2 inhibitor	A10BK, A10BD15, A10BD19, A10BD20, A10BD21, A10BD24
Canagliflozin	A10BK02
Dapagliflozin	A10BK01, A10BD15, A10BD21
Empagliflozin	A10BK03, A10BD19, A10BD20
GLP-1 RAs	A10BJ
Insulin	A10A
Metformin	A10BA02
MRAs	C03A
Other Diuretics	C03 except C03A
Statin	C10AA
Antiplatelet Agents	B01AC04, B01AC06, B01AC22
ACEIs/ARBs	C09
DAAs	J05AP
OAVs	J05AF10, J05AF07, J05AF13, J05AF05, J05AF08, J05AF11
Interferon	L03AB11, L03AB10
Carvedilol	C07AG02
Other NSBBs	C07AA
Procedure	
Abdominal ultrasound	19001C, 19005C, 19009C

© 2026 Chung M-C et al. *JAMA Network Open*.

eTable 2. Sensitivity Analysis of Adverse Outcomes Using Various Propensity Score Trimming Thresholds (IPTW Analysis)

	Crude HR (95% CI)	p value	Model1 HR (95% CI)	p value	Model2 HR (95% CI)	p value
Trimming 1st to 99th percentiles						
ESRD						
SGLT2 inhibitor vs. DPP4 inhibitor	0.34 (0.25 - 0.46)	<.0001	0.33 (0.24 - 0.46)	<.0001	0.35 (0.26 - 0.48)	<.0001
AKI						
SGLT2 inhibitor vs. DPP4 inhibitor	0.65 (0.58 - 0.73)	<.0001	0.65 (0.59 - 0.73)	<.0001	0.66 (0.59 - 0.74)	<.0001
MACE						
SGLT2 inhibitor vs. DPP4 inhibitor	0.66 (0.62 - 0.70)	<.0001	0.67 (0.63 - 0.72)	<.0001	0.68 (0.64 - 0.72)	<.0001
Hepatic decompensation						
SGLT2 inhibitor vs. DPP4 inhibitor	0.67 (0.59 - 0.76)	<.0001	0.67 (0.59 - 0.76)	<.0001	0.67 (0.59 - 0.77)	<.0001
Trimming 2.5th to 97.5th percentiles						
ESRD						
SGLT2 inhibitor vs. DPP4 inhibitor	0.35 (0.25 - 0.47)	<.0001	0.34 (0.25 - 0.47)	<.0001	0.36 (0.26 - 0.49)	<.0001
AKI						
SGLT2 inhibitor vs. DPP4 inhibitor	0.66 (0.59 - 0.74)	<.0001	0.66 (0.59 - 0.74)	<.0001	0.67 (0.60 - 0.74)	<.0001
MACE						
SGLT2 inhibitor vs. DPP4 inhibitor	0.66 (0.62 - 0.70)	<.0001	0.68 (0.64 - 0.72)	<.0001	0.68 (0.64 - 0.72)	<.0001
Hepatic decompensation						
SGLT2 inhibitor vs. DPP4 inhibitor	0.68 (0.59 - 0.77)	<.0001	0.67 (0.59 - 0.77)	<.0001	0.67 (0.59 - 0.76)	<.0001
Trimming 5th to 95th percentiles						
ESRD						
SGLT2 inhibitor vs. DPP4 inhibitor	0.33 (0.24 - 0.45)	<.0001	0.33 (0.24 - 0.45)	<.0001	0.35 (0.25 - 0.48)	<.0001
AKI						
SGLT2 inhibitor vs. DPP4 inhibitor	0.66 (0.59 - 0.73)	<.0001	0.66 (0.59 - 0.73)	<.0001	0.66 (0.59 - 0.74)	<.0001
MACE						
SGLT2 inhibitor vs. DPP4 inhibitor	0.67 (0.63 - 0.72)	<.0001	0.69 (0.65 - 0.74)	<.0001	0.69 (0.65 - 0.73)	<.0001
Hepatic decompensation						
SGLT2 inhibitor vs. DPP4 inhibitor	0.69 (0.60 - 0.79)	<.0001	0.69 (0.60 - 0.78)	<.0001	0.68 (0.60 - 0.78)	<.0001
Trimming 10th to 90th percentiles						

© 2026 Chung M-C et al. *JAMA Network Open*.

ESRD						
SGLT2 inhibitor vs. DPP4 inhibitor	0.36 (0.26 - 0.50)	<.0001	0.36 (0.26 - 0.49)	<.0001	0.38 (0.27 - 0.52)	<.0001
AKI						
SGLT2 inhibitor vs. DPP4 inhibitor	0.67 (0.60 - 0.75)	<.0001	0.67 (0.60 - 0.75)	<.0001	0.67 (0.60 - 0.75)	<.0001
MACE						
SGLT2 inhibitor vs. DPP4 inhibitor	0.68 (0.64 - 0.73)	<.0001	0.69 (0.65 - 0.74)	<.0001	0.69 (0.64 - 0.74)	<.0001
Hepatic decompensation						
SGLT2 inhibitor vs. DPP4 inhibitor	0.72 (0.63 - 0.83)	<.0001	0.72 (0.63 - 0.83)	<.0001	0.73 (0.63 - 0.83)	<.0001

© 2026 Chung M-C et al. *JAMA Network Open*.

eTable 3. Subgroup Hazard Ratios for ESRD, AKI, and MACE Associated with SGLT2 vs. DPP4 Inhibitors (IPTW Analysis)

	ESRD		AKI		MACE	
	Event	HR (95%CI)	Event	HR (95%CI)	Event	HR (95%CI)
Age						
18 to 64	40 vs 184	0.33 (0.23 - 0.48)***	331 vs 698	0.67 (0.58 - 0.77)***	825 vs 1,799	0.69 (0.63 - 0.76)***
65+	20 vs 155	0.37 (0.22 - 0.63)**	237 vs 1,010	0.64 (0.54 - 0.76)***	815 vs 3,333	0.64 (0.58 - 0.70)***
Gender						
Male	48 vs 229	0.37 (0.26 - 0.52)***	422 vs 1,041	0.69 (0.61 - 0.79)***	1,240 vs 3,193	0.69 (0.64 - 0.75)***
Female	12 vs 110	0.29 (0.14 - 0.59)**	146 vs 667	0.63 (0.50 - 0.79)***	400 vs 1,939	0.63 (0.55 - 0.72)***
HTN						
No	20 vs 83	0.44 (0.26 - 0.75)**	191 vs 541	0.68 (0.57 - 0.82)***	583 vs 1,713	0.69 (0.62 - 0.77)***
Yes	40 vs 256	0.31 (0.21 - 0.46)***	377 vs 1,167	0.65 (0.57 - 0.76)***	1,057 vs 3,419	0.66 (0.61 - 0.72)***
HPL						
No	30 vs 181	0.37 (0.24 - 0.57)***	277 vs 981	0.70 (0.59 - 0.82)***	860 vs 3,209	0.65 (0.60 - 0.72)***
Yes	30 vs 158	0.34 (0.21 - 0.53)***	291 vs 727	0.62 (0.53 - 0.73)***	780 vs 1,923	0.69 (0.62 - 0.76)***
CVD						
No	51 vs 299	0.31 (0.22 - 0.44)***	507 vs 1,446	0.65 (0.58 - 0.73)***	1,471 vs 4,336	0.67 (0.63 - 0.72)***
Yes	9 vs 40	0.74 (0.35 - 1.55)	61 vs 262	0.72 (0.51 - 1.02)	169 vs 796	0.65 (0.52 - 0.80)***
CAD						
No	41 vs 281	0.30 (0.21 - 0.44)***	429 vs 1,432	0.60 (0.53 - 0.69)***	1,204 vs 4,168	0.64 (0.59 - 0.69)***
Yes	19 vs 58	0.48 (0.26 - 0.89)*	139 vs 276	0.97 (0.76 - 1.23)	436 vs 964	0.78 (0.68 - 0.89)**
CKD						
No	42 vs 184	0.42 (0.29 - 0.61)***	412 vs 1,173	0.67 (0.58 - 0.76)***	1,288 vs 3,820	0.67 (0.62 - 0.72)***
Yes	18 vs 155	0.22 (0.13 - 0.39)***	156 vs 535	0.62 (0.50 - 0.77)***	352 vs 1,312	0.64 (0.56 - 0.73)***
HBV						
No	52 vs 272	0.39 (0.28 - 0.54)***	457 vs 1,420	0.65 (0.58 - 0.74)***	1,314 vs 4,216	0.66 (0.61 - 0.71)***
Yes	8 vs 67	0.19 (0.09 - 0.40)***	111 vs 288	0.71 (0.55 - 0.91)**	326 vs 916	0.69 (0.60 - 0.80)***
HCV						
No	55 vs 277	0.37 (0.27 - 0.51)***	493 vs 1,378	0.66 (0.59 - 0.75)***	1,407 vs 4,129	0.66 (0.61 - 0.71)***
Yes	5 vs 62	0.21 (0.07 - 0.59)**	75 vs 330	0.65 (0.47 - 0.88)**	233 vs 1,003	0.71 (0.60 - 0.85)**
AMI						
No	58 vs 335	0.34 (0.25 - 0.47)***	546 vs 1,682	0.65 (0.58 - 0.73)***	1,570 vs 5,040	0.66 (0.62 - 0.71)***

© 2026 Chung M-C et al. *JAMA Network Open*.

Yes	2 vs 4	0.32 (0.23 - 0.45)***	22 vs 26	1.16 (0.59 - 2.27)	70 vs 92	0.71 (0.49 - 1.02)
Stroke						
No	52 vs 300	0.67 (0.29 - 1.54)	518 vs 1,505	0.65 (0.58 - 0.73)***	1,511 vs 4,484	0.67 (0.63 - 0.72)***
Yes	8 vs 39	0.35 (0.25 - 0.49)***	50 vs 203	0.75 (0.51 - 1.12)	129 vs 648	0.64 (0.50 - 0.81)**
HF						
No	53 vs 304	0.24 (0.10 - 0.61)**	487 vs 1,555	0.62 (0.55 - 0.70)***	1,361 vs 4,571	0.64 (0.60 - 0.69)***
Yes	7 vs 35	0.34 (0.25 - 0.46)***	81 vs 153	1.12 (0.77 - 1.61)	279 vs 561	0.92 (0.77 - 1.09)
NASH						
No	58 vs 338	0.32 (0.23 - 0.44)***	560 vs 1,702	0.65 (0.58 - 0.73)***	1,628 vs 5,105	0.67 (0.62 - 0.71)***
Yes	2 vs 1	0.34 (0.25 - 0.47)***	8 vs 6	2.32 (0.55 - 9.78)	12 vs 27	0.68 (0.30 - 1.54)
ALC						
No	56 vs 314	0.46 (0.14 - 1.54)	512 vs 1,500	0.66 (0.59 - 0.75)***	1,520 vs 4,608	0.68 (0.64 - 0.73)***
Yes	4 vs 25	0.34 (0.24 - 0.46)***	56 vs 208	0.71 (0.49 - 1.03)	120 vs 524	0.56 (0.44 - 0.71)***
Hypoglycemia						
No	58 vs 324	0.51 (0.16 - 1.59)	549 vs 1,640	0.63 (0.57 - 0.71)***	1,601 vs 4,927	0.65 (0.61 - 0.70)***
Yes	2 vs 15	0.35 (0.25 - 0.47)***	19 vs 68	1.75 (0.91 - 3.38)	39 vs 205	1.19 (0.75 - 1.87)
GLP-1 RAs						
No	59 vs 338	0.33 (0.24 - 0.45)***	563 vs 1,703	0.66 (0.59 - 0.74)***	1,625 vs 5,120	0.67 (0.62 - 0.71)***
Yes	1 vs 1	0.38 (0.26 - 0.54)***	5 vs 5	0.13 (0.01 - 3.85)	15 vs 12	0.41 (0.15 - 1.10)
Insulin						
No	44 vs 272	0.23 (0.13 - 0.42)***	432 vs 1,458	0.67 (0.59 - 0.76)***	1,322 vs 4,506	0.67 (0.62 - 0.72)***
Yes	16 vs 67	0.33 (0.23 - 0.47)***	136 vs 250	0.61 (0.48 - 0.77)***	318 vs 626	0.64 (0.55 - 0.74)***
Metformin						
No	46 vs 284	0.45 (0.23 - 0.87)*	414 vs 1,292	0.68 (0.59 - 0.78)***	1,207 vs 3,834	0.67 (0.62 - 0.72)***
Yes	14 vs 55	0.34 (0.25 - 0.48)***	154 vs 416	0.62 (0.50 - 0.77)***	433 vs 1,298	0.65 (0.57 - 0.74)***
MRAs						
No	55 vs 308	0.40 (0.14 - 1.14)	500 vs 1,502	0.64 (0.57 - 0.72)***	1,468 vs 4,562	0.67 (0.62 - 0.72)***
Yes	5 vs 31	0.35 (0.25 - 0.50)***	68 vs 206	0.81 (0.59 - 1.10)	172 vs 570	0.70 (0.57 - 0.86)**
Other Diuretics						
No	46 vs 259	0.31 (0.17 - 0.58)**	461 vs 1,349	0.65 (0.57 - 0.74)***	1,345 vs 4,119	0.66 (0.62 - 0.71)***
Yes	14 vs 80	0.40 (0.28 - 0.57)***	107 vs 359	0.71 (0.54 - 0.92)*	295 vs 1,013	0.70 (0.60 - 0.81)***
Statin						

© 2026 Chung M-C et al. *JAMA Network Open*.

No	45 vs 252	0.23 (0.12 - 0.43)***	414 vs 1,386	0.66 (0.58 - 0.75)***	1,189 vs 4,213	0.65 (0.60 - 0.70)***
Yes	15 vs 87	0.34 (0.24 - 0.48)***	154 vs 322	0.69 (0.56 - 0.86)**	451 vs 919	0.72 (0.63 - 0.82)***
Antiplatelet Agents						
No	46 vs 288	0.34 (0.17 - 0.69)**	468 vs 1,456	0.66 (0.58 - 0.74)***	1,317 vs 4,363	0.67 (0.62 - 0.72)***
Yes	14 vs 51	0.38 (0.25 - 0.57)***	100 vs 252	0.68 (0.51 - 0.90)**	323 vs 769	0.68 (0.58 - 0.79)***
ACEIs/ARBs						
No	33 vs 198	0.30 (0.19 - 0.48)***	338 vs 1,124	0.61 (0.53 - 0.71)***	1,016 vs 3,427	0.65 (0.60 - 0.70)***
Yes	27 vs 141	0.34 (0.25 - 0.47)***	230 vs 584	0.76 (0.63 - 0.93)**	624 vs 1,705	0.71 (0.64 - 0.80)***
DAAs						
No	60 vs 338	0.33 (0.24 - 0.45)***	568 vs 1,705	0.66 (0.59 - 0.74)***	1,638 vs 5,125	0.66 (0.62 - 0.71)***
Yes	0 vs 1	0.35 (0.25 - 0.48)***	0 vs 3	-	2 vs 7	2.52 (0.65 - 9.78)
OAVs						
No	56 vs 313	0.30 (0.10 - 0.92)*	513 vs 1,601	0.64 (0.57 - 0.72)***	1,494 vs 4,766	0.67 (0.62 - 0.71)***
Yes	4 vs 26	0.34 (0.25 - 0.47)***	55 vs 107	0.88 (0.61 - 1.26)	146 vs 366	0.67 (0.54 - 0.83)**
Interferon						
No	60 vs 339	0.33 (0.24 - 0.45)***	568 vs 1,706	0.66 (0.59 - 0.74)***	1,640 vs 5,128	0.67 (0.62 - 0.71)***
Yes	0 vs 0	-	0 vs 2	-	0 vs 4	-
Carvedilol						
No	56 vs 322	0.35 (0.25 - 0.48)***	547 vs 1,634	0.67 (0.60 - 0.75)***	1,575 vs 4,944	0.67 (0.63 - 0.72)***
Yes	4 vs 17	0.31 (0.09 - 1.12)	21 vs 74	0.41 (0.23 - 0.73)**	65 vs 188	0.66 (0.47 - 0.94)*
Other NSBBs						
No	57 vs 321	0.33 (0.24 - 0.46)***	519 vs 1,551	0.65 (0.57 - 0.73)***	1,503 vs 4,709	0.66 (0.62 - 0.71)***
Yes	3 vs 18	0.46 (0.13 - 1.60)	49 vs 157	0.82 (0.56 - 1.19)	137 vs 423	0.76 (0.60 - 0.95)*

Each outcome event of SGLT2 and DPP4 inhibitor

Adjusted DM Duration, Age, Gender, Comorbidities and Medications

p value < 0.05 : * ; p value < 0.01 : ** ; p value < 0.0001 : ***

© 2026 Chung M-C et al. *JAMA Network Open*.

eFigure 2. Hazard Ratios for MACE and hepatic Decompensation: SGLT2i vs DPP4i in T2DM patients with cirrhosis.

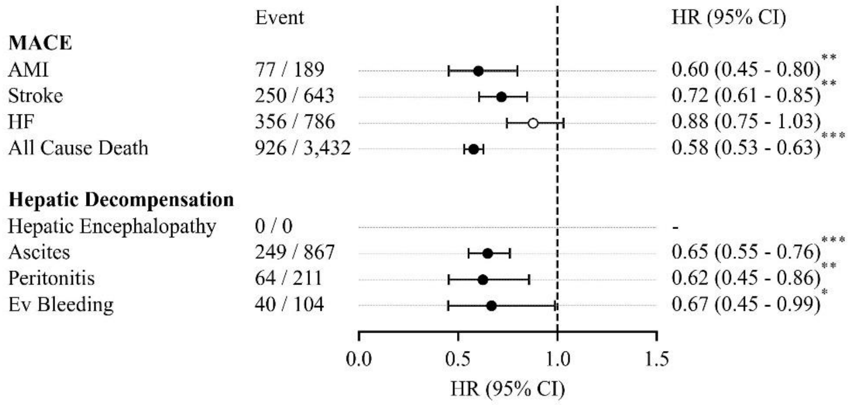

*P<0.05; **P<0.01; ***P<0.001.

© 2026 Chung M-C et al. *JAMA Network Open*.

eTable 4. Baseline Characteristics After Propensity Score Matching in Patients with Type 2 Diabetes Receiving DPP4 or SGLT2 Inhibitors

	PSM Overall	DPP4 inhibitor	SGLT2 inhibitor	SMD	
	(N= 17,794)	(N= 8,897)	(N= 8,897)	Unweighted	Matched
DM Duration (year), Mean ± Std	10.11 ± 6.46	10.10 ± 6.55	10.13 ± 6.38	0.05	0.00
Index Year of receiving SGLT2i or DPP4i, N (%)					
2016	641 (3.60%)	323 (3.63%)	318 (3.57%)	0.28	0.00
2017	1,183 (6.65%)	598 (6.72%)	585 (6.58%)	0.26	0.00
2018	1,541 (8.66%)	830 (9.33%)	711 (7.99%)	0.20	0.04
2019	2,245 (12.62%)	1,201 (13.50%)	1,044 (11.73%)	0.07	0.05
2020	2,855 (16.04%)	1,478 (16.61%)	1,377 (15.48%)	-0.03	0.03
2021	3,304 (18.57%)	1,663 (18.69%)	1,641 (18.44%)	-0.13	0.01
2022	3,150 (17.70%)	1,488 (16.72%)	1,662 (18.68%)	-0.22	-0.05
2023	2,875 (16.16%)	1,316 (14.79%)	1,559 (17.52%)	-0.27	-0.08
Age (year), Mean ± Std	63.19 ± 11.44	63.66 ± 11.76	62.71 ± 11.10		
18 to 24	5 (0.03%)	3 (0.03%)	2 (0.02%)	-0.01	-0.01
25 to 34	97 (0.55%)	52 (0.58%)	45 (0.51%)	-0.02	0.00
35 to 44	894 (5.02%)	434 (4.88%)	460 (5.17%)	-0.10	-0.03
45 to 54	3,049 (17.13%)	1,506 (16.93%)	1,543 (17.34%)	-0.13	-0.03
55 to 64	5,478 (30.79%)	2,614 (29.38%)	2,864 (32.19%)	-0.13	-0.02
65 to 74	5,393 (30.31%)	2,673 (30.04%)	2,720 (30.57%)	-0.01	0.04
75 to 84	2,361 (13.27%)	1,292 (14.52%)	1,069 (12.02%)	0.24	0.04
85+	517 (2.91%)	323 (3.63%)	194 (2.18%)	0.23	-0.01
Gender (Female), N (%)	5,400 (30.35%)	2,819 (31.68%)	2,581 (29.01%)	0.21	0.06
Comorbidity, N (%)					
HTN	10,949 (61.53%)	5,454 (61.30%)	5,495 (61.76%)	0.00	-0.01
HPL	9,509 (53.44%)	4,648 (52.24%)	4,861 (54.64%)	-0.22	-0.05
CVD	1,338 (7.52%)	681 (7.65%)	657 (7.38%)	0.14	0.01
CAD	3,171 (17.82%)	1,517 (17.05%)	1,654 (18.59%)	-0.10	-0.04
CKD	3,387 (19.03%)	1,689 (18.98%)	1,698 (19.09%)	0.04	0.00
HBV	3,839 (21.57%)	1,918 (21.56%)	1,921 (21.59%)	-0.04	0.00

© 2026 Chung M-C et al. *JAMA Network Open*.

HCV	2,345 (13.18%)	1,187 (13.34%)	1,158 (13.02%)	0.12	0.01
AMI	322 (1.81%)	139 (1.56%)	183 (2.06%)	-0.08	-0.04
Stroke	1,025 (5.76%)	530 (5.96%)	495 (5.56%)	0.14	0.02
HF	1,162 (6.53%)	540 (6.07%)	622 (6.99%)	-0.05	-0.04
NASH	232 (1.30%)	111 (1.25%)	121 (1.36%)	-0.04	-0.01
ALC	1,079 (6.06%)	551 (6.19%)	528 (5.93%)	0.07	0.01
Hypoglycemia	202 (1.14%)	105 (1.18%)	97 (1.09%)	0.11	0.01
Medication (Diabetes), N (%)					
GLP-1 Ras	113 (0.64%)	41 (0.46%)	72 (0.81%)	-0.09	-0.04
Insulin	2,145 (12.05%)	1,017 (11.43%)	1,128 (12.68%)	-0.14	-0.04
Metformin	5,459 (30.68%)	2,750 (30.91%)	2,709 (30.45%)	0.00	0.01
Medication (Other), N (%)					
MRAs	917 (5.15%)	472 (5.31%)	445 (5.00%)	0.09	0.01
Other Diuretics	1,791 (10.07%)	901 (10.13%)	890 (10.00%)	0.10	0.00
Statin	5,441 (30.58%)	2,625 (29.50%)	2,816 (31.65%)	-0.21	-0.05
Antiplatelet Agents	2,855 (16.04%)	1,396 (15.69%)	1,459 (16.40%)	-0.08	-0.02
ACEIs/ARBs	6,592 (37.05%)	3,225 (36.25%)	3,367 (37.84%)	-0.10	-0.03
DAAs	13 (0.07%)	6 (0.07%)	7 (0.08%)	0.03	0.00
OAVs	1,854 (10.42%)	918 (10.32%)	936 (10.52%)	-0.05	-0.01
Interferon	0 (0.00%)	0 (0.00%)	0 (0.00%)	-	-
Carvedilol	551 (3.10%)	268 (3.01%)	283 (3.18%)	-0.01	-0.01
Other NSBBs	1,198 (6.73%)	615 (6.91%)	583 (6.55%)	0.05	0.01

HTN: Hypertension; HPL: Hyperlipidemia; CVD: Cerebral Vascular Disease; CAD: Coronary Artery Disease; CKD: Chronic Kidney Disease; HF: Heart Failure; HBV: Hepatitis B Virus Infection; HCV: Hepatitis C Virus Infection; NASH: Nonalcoholic Steatohepatitis; ALC: Alcoholic Liver Cirrhosis; MRAs: Mineralocorticoid receptor antagonists; GLP-1 RAs: GLP-1 Receptor Agonists; OAVs: Oral Antiviral Agents for Chronic Hepatitis B; ONBBs: Other non-selective beta blockers; NSBBs: Non-selective beta-blockers

© 2026 Chung M-C et al. *JAMA Network Open*.

eTable 5. Adjusted Risk of Adverse Renal and Cardiovascular Outcomes Among Patients with Type 2 Diabetes and Liver Cirrhosis Using SGLT2 or DPP4 Inhibitors (propensity score matching)

	Event	PY	IR	Crude HR (95% CI)	p value	Model 1 HR (95% CI)	p value	Model 2 HR (95% CI)	p value
ESRD									
DPP4 inhibitor (N = 8,897)	175	24330.48	7.19	REF		REF		REF	
SGLT2 inhibitor (N = 8,897)	56	24679.55	2.27	0.36 (0.25 - 0.52)	<.0001	0.36 (0.24 - 0.54)	<.0001	0.30 (0.15 - 0.60)	0.0007
AKI									
DPP4 inhibitor (N = 8,897)	785	23674.70	33.16	REF		REF		REF	
SGLT2 inhibitor (N = 8,897)	530	24183.62	21.92	0.68 (0.59 - 0.79)	<.0001	0.68 (0.59 - 0.79)	<.0001	0.66 (0.56 - 0.78)	<.0001
MACE									
DPP4 inhibitor (N = 8,897)	2248	23392.34	96.10	REF		REF		REF	
SGLT2 inhibitor (N = 8,897)	1559	23695.06	65.79	0.65 (0.60 - 0.71)	<.0001	0.66 (0.60 - 0.72)	<.0001	0.63 (0.58 - 0.69)	<.0001
Hepatic decompensation									
DPP4 inhibitor (N = 8,897)	517	24122.57	21.43	REF		REF		REF	
SGLT2 inhibitor (N = 8,897)	351	24348.72	14.42	0.59 (0.50 - 0.71)	<.0001	0.60 (0.50 - 0.72)	<.0001	0.63 (0.51 - 0.78)	<.0001

Incidence Rate is equal to the number of events per 1,000 Person-Year. PY: Person-Year; IR: Incidence Rate
Model 1 Adjusted DM Duration, Age and Gender
Model 2 Adjusted DM Duration, Age, Gender, Comorbidities and Medications

© 2026 Chung M-C et al. *JAMA Network Open*.

eTable 6. Hazard Ratios for AMI, Stroke, and HF Associated with SGLT2 vs. DPP4 Inhibitors by Visit Type (IPTW Analysis)

	SGLT2 inhibitor				DPP4 inhibitor				Crude HR		Adjusted HR	
	N	Event	PY	IR	N	Event	PY	IR	(95% CI)	p value	(95% CI)	p value
Outpatient and Inpatient												
AMI	5,759	45	15,505.60	2.90	7,539	122	24,760.68	4.93	0.54 (0.37 - 0.78)	0.001	0.53 (0.37 - 0.78)	0.0011
STROKE	5,924	210	15,820.51	13.27	7,906	489	25,564.51	19.13	0.76 (0.63 - 0.91)	0.0023	0.76 (0.64 - 0.92)	0.0039
HF	5,881	167	15,780.30	10.58	7,820	403	25,388.65	15.87	0.70 (0.57 - 0.85)	0.0004	0.68 (0.56 - 0.83)	0.0002
Outpatient												
AMI	5,742	28	15,472.28	1.81	7,486	69	24,625.77	2.80	0.56 (0.35 - 0.89)	0.0154	0.55 (0.35 - 0.88)	0.0132
STROKE	5,849	135	15,692.51	8.60	7,764	347	25,264.26	13.73	0.70 (0.56 - 0.87)	0.0011	0.70 (0.56 - 0.88)	0.0018
HF	5,825	111	15,658.00	7.09	7,689	272	25,077.30	10.85	0.71 (0.56 - 0.91)	0.0066	0.70 (0.55 - 0.90)	0.0057
Inpatient												
AMI	5,743	29	15,470.08	1.87	7,506	89	24,675.76	3.61	0.47 (0.30 - 0.75)	0.0013	0.47 (0.30 - 0.75)	0.0014
STROKE	5,813	99	15,591.63	6.35	7,634	217	24,959.24	8.69	0.79 (0.61 - 1.02)	0.0717	0.79 (0.60 - 1.03)	0.0822
HF	5,781	67	15,565.94	4.30	7,567	150	24,807.30	6.05	0.70 (0.51 - 0.95)	0.021	0.68 (0.49 - 0.92)	0.0136

Incidence Rate is equal to the number of events per 1,000 Person-Year. PY: Person-Year; IR: Incidence Rate
Adjusted DM Duration, Age, Gender, Comorbidities and Medications

© 2026 Chung M-C et al. *JAMA Network Open*.

eTable 7. Hazard Ratios for ESRD, AKI, and MACE Associated with SGLT2 vs. DPP4 Inhibitors by CKD Stage (IPTW Analysis)

	SGLT2 inhibitor				DPP4 inhibitor				Crude HR		Adjusted HR	
	N	Event	PY	IR	N	Event	PY	IR	(95% CI)	p value	(95% CI)	p value
ESRD												
Non CKD	8,425	45	23,253.52	1.94	12,416	225	40,789.74	5.52	0.37 (0.25 - 0.53)	<.0001	0.36 (0.25 - 0.51)	<.0001
CKD Stage I & II	356	0	768.25	0.00	329	9	849.34	10.60	-	-	-	-
CKD Stage III	580	12	1,115.18	10.76	1,079	59	2,916.59	20.23	0.55 (0.27 - 1.10)	0.0902	0.61 (0.31 - 1.22)	0.1606
CKD Unspecified	328	3	959.83	3.13	746	46	2,393.74	19.22	0.11 (0.03 - 0.43)	0.0015	0.10 (0.02 - 0.45)	0.0028
AKI												
Non CKD	8,425	452	22,842.12	19.79	12,416	1,324	39,450.79	33.56	0.67 (0.59 - 0.77)	<.0001	0.64 (0.57 - 0.73)	<.0001
CKD Stage I & II	356	33	734.02	44.96	329	43	804.79	53.43	1.06 (0.65 - 1.75)	0.8092	1.05 (0.60 - 1.85)	0.863
CKD Stage III	580	44	1,080.81	40.71	1,079	200	2,748.26	72.77	0.48 (0.33 - 0.69)	<.0001	0.51 (0.35 - 0.75)	0.0006
CKD Unspecified	328	39	916.76	42.54	746	141	2,287.23	61.65	0.70 (0.47 - 1.04)	0.0809	0.85 (0.55 - 1.31)	0.4563
MACE												
Non CKD	8,425	1,391	22,393.61	62.12	12,416	4,192	38,919.90	107.71	0.67 (0.63 - 0.72)	<.0001	0.66 (0.61 - 0.71)	<.0001
CKD Stage I & II	356	60	722.97	82.99	329	89	811.35	109.69	0.92 (0.63 - 1.33)	0.6402	0.91 (0.59 - 1.41)	0.6741
CKD Stage III	580	107	1,049.61	101.94	1,079	466	2,737.42	170.23	0.61 (0.47 - 0.78)	<.0001	0.61 (0.48 - 0.78)	<.0001
CKD Unspecified	328	82	895.73	91.55	746	385	2,272.07	169.45	0.56 (0.43 - 0.74)	<.0001	0.70 (0.53 - 0.93)	0.0142
Hepatic decompensation												
Non CKD	8,425	324	22,946.20	14.12	12,416	1,038	39,999.99	25.95	0.67 (0.58 - 0.77)	<.0001	0.66 (0.57 - 0.76)	<.0001
CKD Stage I & II	356	11	754.77	14.57	329	18	843.78	21.33	0.76 (0.35 - 1.66)	0.4928	1.03 (0.41 - 2.63)	0.9464
CKD Stage III	580	15	1,112.79	13.48	1,079	95	2,893.65	32.83	0.44 (0.24 - 0.81)	0.0081	0.43 (0.24 - 0.80)	0.0071
CKD Unspecified	328	14	947.83	14.77	746	81	2,400.55	33.74	0.52 (0.27 - 1.02)	0.0559	0.59 (0.32 - 1.08)	0.0846

Incidence Rate is equal to the number of events per 1,000 Person-Year. PY: Person-Year; IR: Incidence Rate
Adjusted DM Duration, Age, Gender, Comorbidities and Medications

© 2026 Chung M-C et al. *JAMA Network Open*.
