# Supplementary material for: SGLT2 Inhibitor Use and Cardiorenal Outcomes in Type 2 Diabetes With Liver Cirrhosis
Source: JAMA Netw Open. 2026 Feb 23;9(2):e2560429. doi: 10.1001/jamanetworkopen.2025.60429 (PMC12930281; doi:10.1001/jamanetworkopen.2025.60429)
Supplement: Supplement 2. — Data Sharing Statement [file jamanetwopen-e2560429-s002.pdf]

Data Sharing Statement

Chung. SGLT2 Inhibitor Use and Cardiorenal Outcomes in Type 2 Diabetes With Liver Cirrhosis. *JAMA Netw Open*. Published February 23, 2026.
doi:10.1001/jamanetworkopen.2025.60429

Data
Data available: No

Additional Information
Explanation for why data not available: Data are available from the National Health Insurance Research Database (NHIRD) published by the Taiwan Ministry of Health and Welfare. Due to legal restrictions imposed by the government of Taiwan in relation to the "Personal Information Protection Act", data cannot be made publicly available. Requests for data can be sent as a formal proposal to the Health and Welfare Data Science Center, Taiwan.
